# Factor VIII antibody immune complexes modulate the humoral response to factor VIII in an epitope-dependent manner

**DOI:** 10.3389/fimmu.2023.1233356

**Published:** 2023-08-31

**Authors:** Glaivy Batsuli, Jasmine Ito, Elizabeth S. York, Courtney Cox, Wallace Baldwin, Surinder Gill, Pete Lollar, Shannon L. Meeks

**Affiliations:** ^1^ Department of Pediatrics, Emory University, Atlanta, GA, United States; ^2^ Aflac Cancer and Blood Disorders Center of Children’s Healthcare of Atlanta, Atlanta, GA, United States

**Keywords:** B-cell epitope, factor VIII, hemophilia, humoral response, immune complex

## Abstract

**Introduction:**

Soluble antigens complexed with immunoglobulin G (IgG) antibodies can induce robust adaptive immune responses *in vitro* and in animal models of disease. Factor VIII immune complexes (FVIII-ICs) have been detected in individuals with hemophilia A and severe von Willebrand disease following FVIII infusions. Yet, it is unclear if and how FVIII-ICs affect antibody development over time.

**Methods:**

In this study, we analyzed internalization of FVIII complexed with epitope-mapped FVIII-specific IgG monoclonal antibodies (MAbs) by murine bone marrow-derived dendritic cells (BMDCs) *in vitro* and antibody development in hemophilia A (FVIII^-/-^) mice injected with FVIII-IC over time.

**Results:**

FVIII complexed with 2-116 (A1 domain MAb), 2-113 (A3 domain MAb), and I55 (C2 domain MAb) significantly increased FVIII uptake by BMDC but only FVIII/2-116 enhanced antibody titers in FVIII^-/-^ mice compared to FVIII alone. FVIII/4A4 (A2 domain MAb) showed similar FVIII uptake by BMDC to that of isolated FVIII yet significantly increased antibody titers when injected in FVIII^-/-^ mice. Enhanced antibody responses observed with FVIII/2-116 and FVIII/4A4 complexes *in vivo* were abrogated in the absence of the FVIII carrier protein von Willebrand factor.

**Conclusion:**

These findings suggest that a subset of FVIII-IC modulates the humoral response to FVIII in an epitope-dependent manner, which may provide insight into the antibody response observed in some patients with hemophilia A.

## Introduction

Hemophilia A is an inherited bleeding disorder characterized by a deficiency of coagulation protein factor VIII (FVIII). Individuals with hemophilia A require intravenous FVIII infusions to treat and prevent bleeding events. Despite intravenous administration of this soluble antigen at nanomolar concentrations, the formation of high-affinity neutralizing antibodies, called inhibitors, occurs in approximately 30% of individuals with severe hemophilia A (1). Inhibitor development renders FVIII infusions ineffective and ultimately results in reduced quality of life, increased cost of care, and worsened disease mortality (2, 3). FVIII is a large glycoprotein consisting of six primary domains (A1, A2, B, ap, A3, C1, and C2). In individuals and murine models of hemophilia A, the immune response to FVIII is a CD4^+^ T cell-dependent process initiated by presentation of FVIII peptides by antigen-presenting cells (APCs) (4–7). Although the A2 and C2 domains are considered the immunodominant domains, a polyclonal response consisting of neutralizing and non-neutralizing antibodies targeting functional and non-functional FVIII epitopes has been described in patients with congenital and acquired hemophilia A and in hemophilia A mice (8–12). A recent prospective cohort study evaluating the antibody profile of 23 previously untreated pediatric patients with severe hemophilia A observed that 30% of participants developed persistent inhibitors and an additional 39% developed non-neutralizing antibodies during the first 50 exposure days to recombinant FVIII (13).

Soluble antigens, such as ovalbumin (OVA), complexed with their cognate immunoglobulin G (IgG) antibody induce more efficient T-cell proliferation than the antigen alone *in vivo* in a dendritic cell (DC)-dependent manner (14). Affinity-matured IgM, IgA, and IgE are also capable of enhancing antibody responses to protein antigens, and thus all Ig subclasses can ultimately influence the antibody response to those antigens (15, 16). However, IgG can distinctly suppress antibody responses to an antigen, as in the case of erythrocytes, via antibody-mediated immune suppression, which is a mechanism that has been exploited as a therapeutic agent in the setting of hemolytic disease of the newborn (17, 18). Hartholt et al. (19) described enhanced internalization of FVIII complexed with polyclonal anti-FVIII IgGs by bone marrow-derived dendritic cells (BMDCs) from C57BL/6 mice when compared to FVIII alone. The role of the A2 and C2 domains in FVIII endocytosis by APCs using monoclonal antibodies (MAbs) identified the contribution of the C2 domain to FVIII endocytosis (20). Additional studies elucidated the C1 domain as the primary modulator of FVIII internalization by human- and murine-derived DCs and macrophages (20–23). However, the contribution of the full spectrum of FVIII domains on FVIII internalization by BMDC derived from FVIII-deficient mice has not been extensively investigated.

Conventional DCs are a subset of APCs adept at presenting peptides to T cells via major histocompatibility complex (MHC) class II molecules resulting in antibody formation (14, 24). Moreover, antigen reexposure in mice primed with immune complexes efficiently induces plasma cell memory responses (25). The Fcγ receptor (FcγR) has been implicated as the mediator of enhanced FVIII internalization when complexed with polyclonal anti-FVIII IgG (19, 26). DCs in mice express activating FcγRI and FcγRIII and the inhibitory FcγRIIb, which have different binding affinities for IgG isotypes versus IgG-containing immune complexes (27). FcγRI exhibits a high binding affinity for monomeric IgG, specifically murine IgG2a (28). However, IgG1, IgG2a, and IgG2b complexed with antigen are capable of engaging and crosslinking lower-affinity FcγRIIb and FcγRIII resulting in potent DC and T-cell activation *in vivo* (14, 28, 29).

Circulating FVIII immune complexes (FVIII-ICs) have been detected in individuals with hemophilia A and severe von Willebrand disease with and without inhibitors following infusion of FVIII-containing concentrates (30–32). In individuals with hemophilia A and persistent inhibitors, immune tolerance induction (ITI) consisting of frequent high-dose FVIII infusions remains the primary strategy for inhibitor eradication and restoration of FVIII tolerance. Yet, successful tolerance is only achieved in 70% of individuals, and 5%–23% of these individuals experience inhibitor relapse within 5 years (33–35). Werwitzke et al. (36) hypothesized that the presence of FVIII-IC may contribute to poorer responses to ITI. However, the role of FVIII-IC in *de novo* antibody development over time and on ITI outcomes remains undefined. Here, we utilize a spectrum of clinically relevant, epitope-mapped, FVIII-specific IgG MAbs to evaluate the role of FVIII-IC on FVIII endocytosis by BMDC and antibody responses in two murine models of hemophilia A. We demonstrate that a subset of epitope-specific FVIII-ICs alters FVIII internalization by BMDC *in vitro* and antibody titers *in vivo* through the FcγR. These findings suggest that FVIII-ICs contribute to the FVIII inhibitor response in an epitope-dependent manner.

## Materials and methods

### Materials

#### Mice

Exon 16-disrupted hemophilia A mice (E16 FVIII^-/-^ mice) on a mixed C57BL/6 (70%) and 129S4 (30%) background were originally obtained from Leon Hoyer (American Red Cross, Holland Laboratory) then backcrossed for >10 generations onto >97% C57BL/6 background (37). FVIII/VWF double-knockout mice (FVIII^-/-^/VWF^-/-^ mice) were generated by crossing E16 FVIII^-/-^ mice with VWF^-/-^ mice on a 100% C57BL/6 background that were obtained as a generous gift from Denisa Wagner (38).

#### Reagents

Murine-derived anti-human FVIII MAbs were purified from hybridomas as previously described (10, 23, 39–41). The characteristics of MAbs utilized in these studies are summarized in **Table 1**. Isotype control IgG1 MAb (anti-factor IX antibody GMA-138) was purchased from Green Mountain Antibodies (Burlington, VT, USA). Isotype control IgG2a antibody C1.18.2 and IgG2b antibody LTF-2 were purchased from Bio X Cell (Lebanon, NH, USA). Fluorophore-conjugated antibodies Pac Blue CD11c (N418), CD11b (M1/70.15), Live/dead fixable near-IR cell stain, APC/Cy7 CD3 (17A2), PE B220 (RA3-6B2), FITC CD80/86 (16-10A1/GL-1), PE CD40 (3/23), and PE MHCII (M5/114.15.2) were purchased from BioLegend (Dedham, MA, USA) or BD Biosciences (San Jose, CA, USA). Alexa Fluor 647-conjugated ovalbumin (OVA) was purchased from Thermo Fisher Scientific (Waltham, MA, USA). Rat anti-mouse CD16 (FcγRIII)/CD32 (FcγRIIb) MAb 2.4G2 was purchased from BD Biosciences (San Jose, CA, USA). Chinese hamster ovary (CHO)-derived full-length recombinant FVIII (FL FVIII; Takeda, Deerfield, IL, USA) was used for BMDC uptake studies following labeling with DyLight 650 (DyL650) dye using an NHS ester kit from Thermo Fisher Scientific (Waltham, MA, USA). B domain-deleted FVIII (BDD FVIII) was expressed and purified as previously described (46–48). Citrated pooled normal plasma (FACT) and FVIII-deficient plasma were purchased from George King Biomedical (Overland Park, KS, USA). All other materials were reagent grade or are described in the cited literature.

**Table 1:** Characteristics of FVIII MAbs.

FVIII MAb	FVIII Domain	IgG Subclass	Inhibitory Titer (BU/mg IgG)	IC50 VWF Binding (μg/mL)	MAb Critical B Cell Binding Epitopes (23, 39–43)
2-116	A1	IgG2a	<1	>10	E11-D15, E53-A78
4A4	A2	IgG2a	40,000	>10	D403-H444
2-54	A2	IgG1	34,000	>10	E604-R740
M2003	B	IgG1	<1	>10	NA
M2005	B	IgG1	<1	>10	NA
F147	A3	IgG1	7,100	>10	S1690-W1817
2-113	A3	IgG1	156	>10	K1818-Y1916
F156	C1	IgG1	7	>10	S2063-I2071, N2129-K2136
M6143	C1	IgG1	180	0.6	S2063-I2071, N2129-K2136
B136	C1	IgG2a	700	0.4	A2077-I2084
3E6	C2	IgG2a	41	0.6	D2187, K2207, H2211, L2212, and Q2213
I89	C2	IgG2a	1,900	0.02	M2199, F2200
1B5	C2	IgG2a	930	0.05	F2196, T2197, N2198, F2200, T2202, R2220, Q2222, N2225, E2228, K2239, L2252, S2254, H2315, and Q2316
3D12	C2	IgG2b	2,600	0.04	Y2195, F2196, N2198, M2199, F2200, T2202, R2220, N2224, N2225, E2228, K2249, S2250, L2251, L2252, T2253, S2254, H2309, and Q2316
2-77	C2	IgG2a	25,000	>10	R2220, N2225, E2228, K2239, H2269, L2273, V2280, R2307, and H2309
B9	C2	IgG2a	31,000	>10	V2223 and K2227
I55	C2	IgG1	10,000	0.02	N2225, E2228, L2273, R2307, and H2309

BU, Bethesda units; IC50, 50% inhibitory concentration; MAb, monoclonal antibody; NA, not available; VWF, von Willebrand factor.

### BMDC generation and endocytosis assay

Murine BMDCs were generated as previously described (22, 49). Briefly, femurs and tibias from euthanized FVIII^-/-^ mice between 8 and 12 weeks of age were harvested, and the bone marrow was flushed with Hank’s balanced salt solution (HBSS). BM cells were washed with HBSS and underwent red cell lysis followed by additional washings with RPMI-1640 containing 10% fetal bovine serum, 100 U/mL penicillin, 100 μg/mL streptomycin, and 2 mM L-glutamine. BM cells were seeded at 2 × 10^6^ cells in 100-mm dishes with 20 ng/mL recombinant mouse granulocyte macrophage colony-stimulating factor (GM-CSF). On day 6, cells were harvested, counted, and washed with serum-free Iscove’s Modified Dulbecco’s Medium prior to treatment with FVIII-IC.

To evaluate FVIII endocytosis, BMDCs were treated with 10 nM DyL650-rFVIII and 80 nM MAb for 30 min at 37°C in serum-free medium. These concentrations are equivalent to 2 µg DyL650-rFVIII and 12 µg MAb. In experiments with FcγR blockade, BMDCs were incubated with 1 µg/mL or 5 µg/mL of MAb 2.4G2 per 1 × 10^6^ BMDC for 15 min at 4°C prior to treatment with FVIII-IC. Following incubation, cells were washed with phosphate buffered saline solution containing 0.5% bovine serum albumin (BSA), stained, and fixed with 1% paraformaldehyde. FVIII internalization by BMDC normalized to untreated/stained BMDC was analyzed by flow cytometry using an LSRII, Cytek Aurora, or ImageStream X Mark II flow cytometer in the Emory University Pediatrics/Winship Cancer Institute Flow Cytometry Core. BMDC surface staining with CD80/86, CD40, and MHC class II antibodies was utilized to determine BMDC maturation after FVIII uptake. BMDC purity and analysis of immature BMDC phenotype were determined utilizing CD3, B220, CD40, CD80/86, and CD11c antibodies and compared to BMDC matured with lipopolysaccharide.

### Sedimentation velocity analytical centrifugation of FVIII-IC

Formation of FVIII-IC for a select group of MAbs with FL FVIII and BDD FVIII was determined by sedimentation velocity analytical centrifugation (SV AUC). SV analysis was performed immediately after a quick thaw of FVIII and MAb samples from -80°C and mixing of FL FVIII and BDD FVIII at a 4-fold to 8-fold molar excess of MAbs. Experiments were performed at 105,000g at 20°C in a Beckman Coulter Proteome Lab XLI analytical centrifuge as previously described (50). Scans were performed at an absorbance of 280 nm in continuous mode at a radial spacing of 0.003 cm. Data were acquired at ~4-min intervals and analyzed with SEDFIT version 16.36 (https://sedfitsedphat.nibib.nih.gov/) using the continuous *c(s)* distribution model. Sedimentation coefficients are reported as (s_w_)_20w_ values, the signal-average sedimentation coefficient adjusted to the standard condition of 20°C in solvent water. SV graphs were plotted using GUSSI version 1.2.1 (51).

### Mouse immunizations with FVIII-IC

FVIII^-/-^ mice were immunized with four weekly retro-orbital injections of 0.1 µg of BDD FVIII ± 1 µg MAb followed by a boost injection of 0.2 µg FVIII ± 2 µg MAb 1 week later for a “low-dose” FVIII regimen. One week after the boost injection, mice were euthanized for plasma collection by cardiac puncture. Utilizing a separate “high-dose” FVIII regimen, FVIII^-/-^ or FVIII^-/-^/VWF^-/-^ mice were immunized with four weekly injections of 1 µg BDD FVIII ± 10 µg MAb followed by a boost dose of 2 µg FVIII ± 20 µg MAb 1 week later. In the high-dose FVIII regimen, plasma samples were collected 2 weeks following the boost injection in FVIII^-/-^ mice to account for murine IgG half-life of 6–8 days and expected higher circulating residual MAbs from injections that could interfere with ELISA and Bethesda titer analyses (52). To further account for the potential effect of residual injected MAbs on antibody titers, plasma samples from mice injected with anti-FVIII MAb without FVIII were analyzed. In both the “low-dose” and “high-dose” FVIII regimens, a 1:10 ratio of FVIII to MAb was used. A separate cohort of FVIII^-/-^ mice was immunized as described above per the “high-dose” FVIII regimen but was administered a 1:1 FVIII-to-MAb ratio (i.e., 1 µg FVIII:1 µg MAb).

### FVIII antibody detection and inhibitor assays

Plasma anti-FVIII IgG titers following FVIII-IC injections were determined by ELISA as previously described (42). Briefly, 96-well high-binding ELISA plates were coated with 1.5 µg/ml BDD FVIII in 20 mM Bicine and 2 mM CaCl_2_ overnight at 4°C. Plates were washed with 20 mM HEPES, 0.15 M NaCl, 2 mM CaCl_2_, 0.05% Tween-20, and 0.05% sodium azide (wash buffer) and blocked with wash buffer + 2% BSA overnight at 4°C. Mouse plasma starting at 1/20 dilution was serially diluted 3.5-fold and incubated on ELISA plates for 1 h. Plasma samples were analyzed in duplicate wells. FVIII-specific IgG antibodies in mouse plasma were captured by goat-anti-mouse IgG conjugated to alkaline phosphatase at 1:500 dilution and detected by *p*-nitrophenyl-phosphate substrate. The reaction was quenched at 20 min with 0.4 M NaOH. ELISA plates were measured at A_405_ and fitted to a 4-parameter logistic equation. The ELISA titer was determined by the A_405_ at 0.3 on the fitted curve. The inhibitory titer was determined by the Nijmegen Bethesda assay using citrated pooled normal human plasma as the FVIII source as previously described (53).

### Plasma domain mapping assay

ELISAs were performed utilizing human BDD FVIII, porcine FVIII, and porcine FVIII constructs with single human FVIII domain substitution (i.e., porcine FVIII with single human A1, A2, A3, ap, C1, or C2 domain substitution) to determine domain specificity of anti-FVIII antibodies produced by immunized FVIII^-/-^ mice (10). Briefly, ELISA plates were coated with BDD FVIII, porcine FVIII, and porcine FVIII with single human FVIII domain substitution constructs. Plasma from FVIII^-/-^ mice starting at 1/20 dilution was serially diluted 2-fold on ELISA plates. Anti-FVIII antibodies were captured, detected, and measured similarly to the methods outlined in the FVIII antibody detection assay. The predominant FVIII domains recognized by FVIII antibodies generated in FVIII^-/-^ mice injected with FVIII-IC were determined by the ELISA titer.

### Statistical analysis

Data are presented as mean ± standard deviation (SD) for *in vitro* studies and median with Q1 and Q3 interquartile ranges (IQRs) for *in vivo* studies. Differences in FVIII internalization by BMDCs were determined by one-way ANOVA with Dunnett’s correction for multiple comparisons. Differences in ELISA and Bethesda titers were determined by the nonparametric Mann–Whitney U test. A *P* value<0.05 was considered statistically significant. Statistical analyses were performed with Prism 6.0 (GraphPad Software, La Jolla, CA, USA).

## Results

### FVIII-ICs differentially alter FVIII endocytosis by BMDC

**Figure 1:**
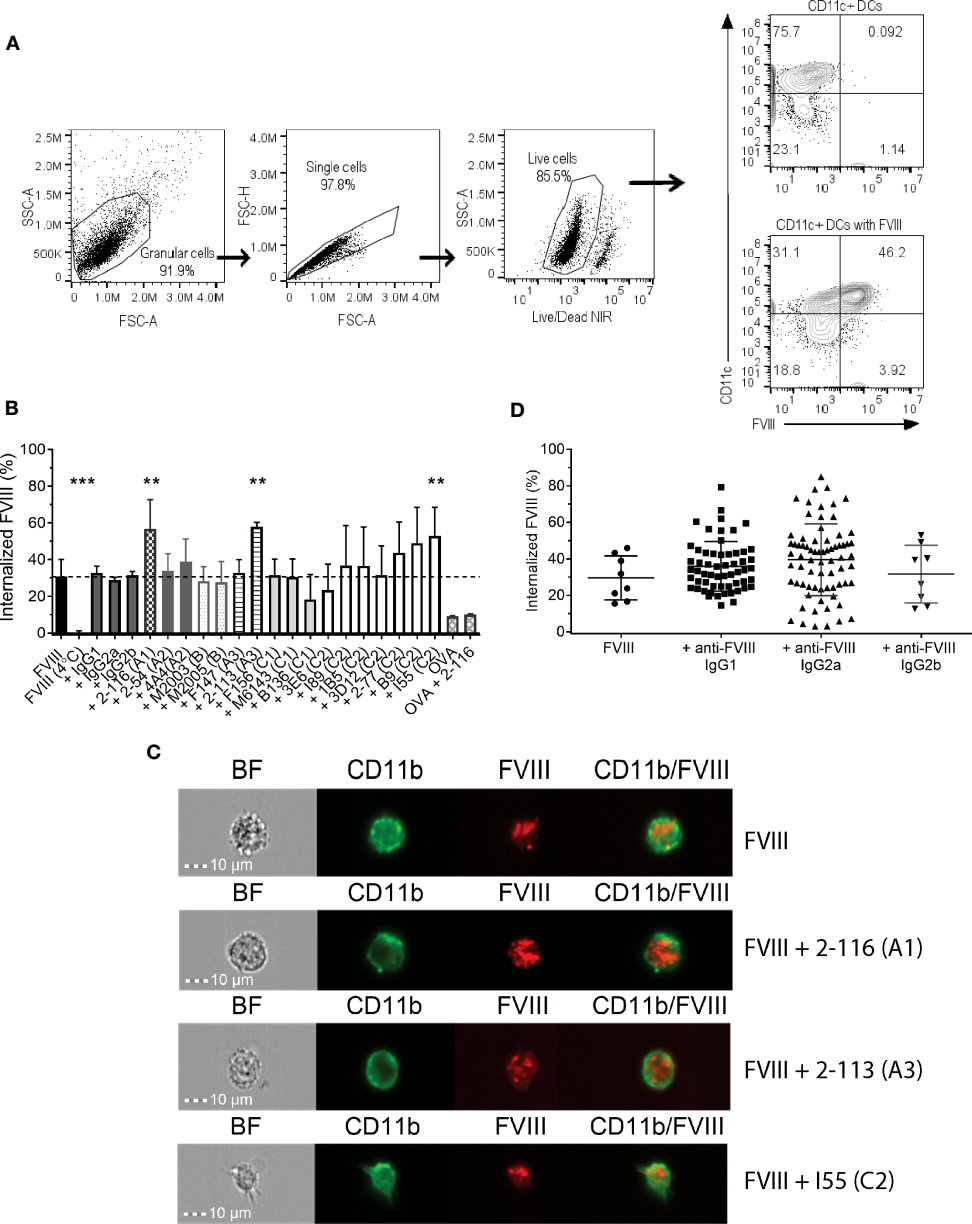
Endocytosis of FVIII-IC by BMDC from FVIII^-/-^ mice. Schematic of flow analysis of CD11c^+^ BMDC in the absence and presence of FVIII **(A)**. Summary of FVIII uptake by BMDC of 10 nM DyL650-conjugated FVIII in complex with 80 nM MAbs recognizing each FVIII domain and non-FVIII-binding IgG isotype controls are shown **(B)**. Internalized FVIII percentages were normalized to the fluorescence of stained BMDC treated with serum-free medium alone. FVIII incubated with BMDC at 4°C served as a negative control to demonstrate differences between FVIII internalization (37°C) and the absence of FVIII internalization (4°C). The dashed horizontal line represents FVIII uptake by BMDC treated with FVIII alone at 37°C. Representative images of FVIII and FVIII-IC internalization by BMDC using ImageStream flow cytometry are shown **(C)**. ImageStream images demonstrate BMDC morphology [Brightfield (BF)] in addition to CD11b, FVIII, and composite CD11b/FVIII staining of BMDC incubated with representative FVIII-IC groups FVIII/2-116 (A1 domain MAb), FVIII/2-113 (A3 domain MAb), and FVIII/I55 (C2 domain MAb) for 30 min at 37°C. Internalized FVIII percentages of all FVIII-IC by IgG isotype are shown with exclusion of non-FVIII IgG isotype controls **(D)**. Measurements of uptake for each FVIII-IC group in panels **(B, C)** were performed in two replicate measurements on three independent/separate days (i.e., total of six replicates). FVIII uptake in the absence of MAbs was performed in two replicate measurements on 4 independent/separate days (i.e., total of eight replicates), corresponding with each day of FVIII-IC uptake testing. Differences in FVIII-IC uptake were compared to FVIII uptake in the absence of MAbs by one-way ANOVA with Dunnett’s correction for multiple comparisons. **P<0.01 and ***P< 0.001. ANOVA, analysis of variance; BF, Brightfield; BMDC, bone marrow derived dendritic cell; DCs, dendritic cells; FVIII, factor VIII; FVIII-IC, FVIIII-immune complexes; IgG, immunoglobulin G; MAbs, monoclonal antibodies.

Prior studies have shown that C1 and C2 domain antibodies affect FVIII endocytosis by DC (20–22). In this study, we utilized a panel of 17 murine-derived anti-human FVIII MAbs directed against each FVIII domain (**Table 1**) to evaluate the effect of FVIII-IC on FVIII endocytosis by BMDC (**Figure 1**). These MAbs are representative of a large repertoire of high-binding affinity IgG MAbs previously characterized (10, 23, 39–41, 44). The gating strategy of flow analysis of CD11c^+^ DC with and without FVIII is depicted (**Figure 1A**). Most FVIII-ICs containing A2, B, or C2 domain MAbs demonstrated similar FVIII uptake to FVIII alone (**Figure 1B**; **Supplementary Figure S1**). FVIII-IC with A1 MAb 2-116 and A3 MAb 2-113 significantly increased FVIII uptake by BMDC by 92% (FVIII/2-116 internalized 56.7% ± 15.9%, *P* = 0.002) and 95% (FVIII/2-113 internalized 57.7% ± 2.6%, *P* = 0.02), respectively, compared to that of FVIII (FVIII internalized 29.6% ± 12.1%). Additionally, C2 MAb I55 increased FVIII uptake by 79% (FVIII/I55 internalized 56.7% ± 15.6%, *P* = 0.01). C1 MAb B136 reduced FVIII uptake by 38%, although this did not reach statistical significance (FVIII/B136 internalized 18.3% ± 13.6%, *P* = 0.65). FVIII incubated with BMDC at 4°C abrogated FVIII uptake as expected. Coincubation of FVIII with non-FVIII-binding isotype control IgG1, IgG2a, and IgG2b antibodies showed similar uptake to that of FVIII alone. This indicates that differences in uptake of some FVIII-IC is due to the binding of FVIII-specific MAbs to FVIII and not excess IgG. OVA with and without FVIII-specific MAb 2-116 showed similarly reduced uptake by BMDC, which provides further evidence that the composition of the FVIII-IC modulates FVIII uptake and is less likely influenced by excess IgG. Lastly, internalization of FVIII-IC by BMDC as opposed to BMDC surface binding of FVIII-IC was also confirmed with a subset of FVIII-IC using ImageStream flow cytometry (**Figure 1C**). Taken together, these findings suggest that epitopes within the A1, A3, and C-terminus of the C2 domain contribute to FVIII recognition by BMDC.

To evaluate other antibody-specific factors that could account for differences in FVIII uptake by BMDC between FVIII-IC, we analyzed FVIII-IC uptake by IgG isotype. The composition of the 17 MAbs tested consisted of eight IgG1 (47%), eight IgG2a (47%), and one IgG2b (6%). There were no differences in BMDC internalization of FVIII-IC by IgG isotype (**Figure 1D**). Thus, differences in FVIII uptake by the 18 FVIII-IC could not be solely attributed to the IgG isotype and are likely secondary to MAb epitope specificity. However, it is important to acknowledge the limited number of anti-FVIII IgG2b MAbs available in this analysis and its potential impact on the differences in FVIII-IC uptake by BMDC when analyzed by IgG isotype. Despite differences in the uptake of FVIII-IC, incubation of FVIII or FVIII-IC with BMDC *in vitro* did not result in maturation of BMDC (**Supplementary Figure S2**). This is consistent with prior studies utilizing murine and human-derived DC *in vitro* (19, 54).

### Anti-FVIII MAbs form 1:1 immune complexes with FVIII

**Figure 2:**
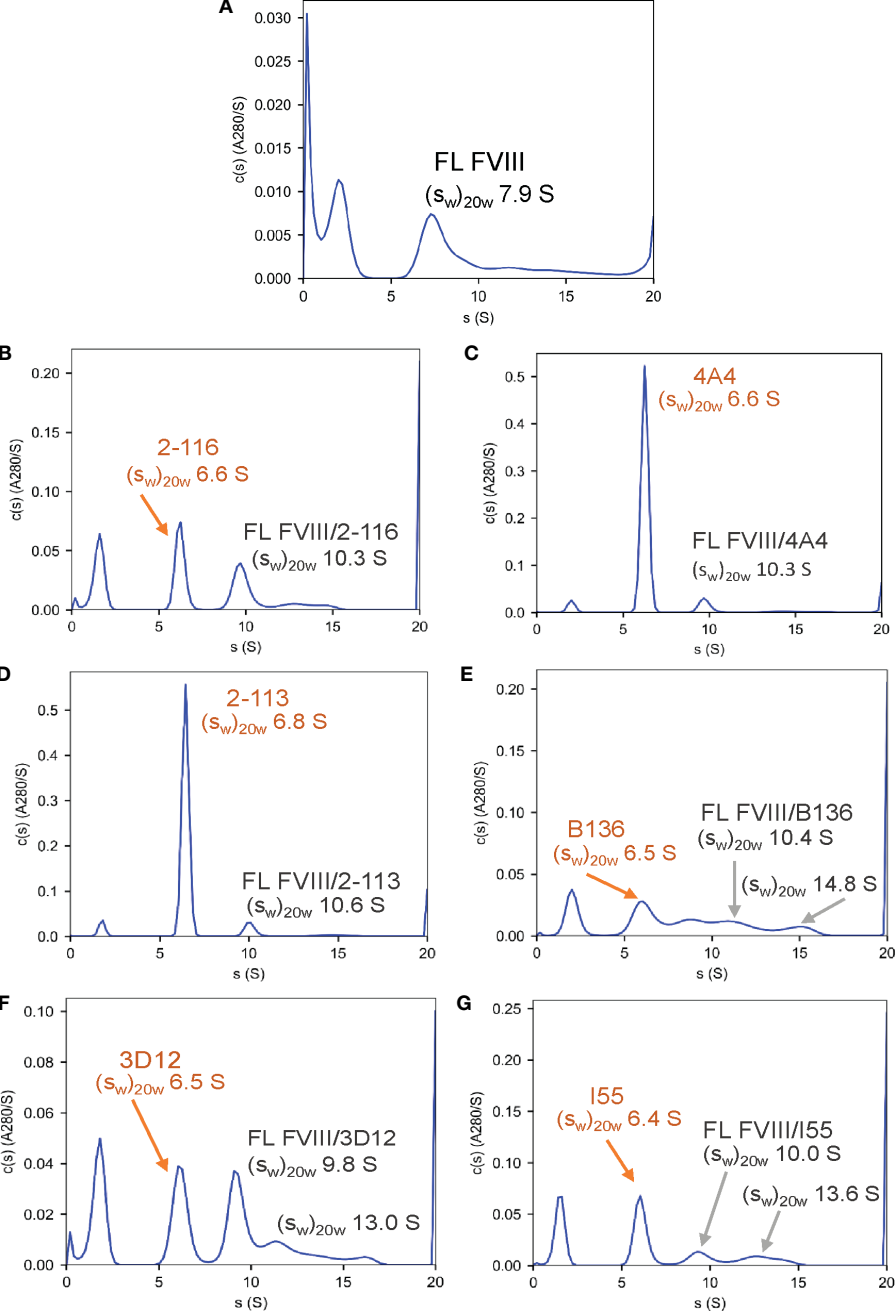
Characterization of FVIII-IC with FL FVIII by SV AUC. SV AUC was performed as described in *Materials and Methods*. Signal-average sedimentation coefficients, adjusted to the standard condition, ${\left(s_{w}\right)}_{20,w}$ , of 20°C in solvent water were estimated by integration of the continuous c(s) distributions in SEDFIT for samples containing FL FVIII alone **(A)**, FVIII/2-116 **(B)**, FVIII/4A4 **(C)**, FVIII/2-113 **(D)**, FVIII/B136 **(E)**, FVIII/3D12 **(F)**, and FVIII/I55 **(G)**. FVIII, factor VIII; FVIII-IC, FVIII-immune complexes; SV AUC, sedimentation velocity analytical centrifugation.

**Figure 3:**
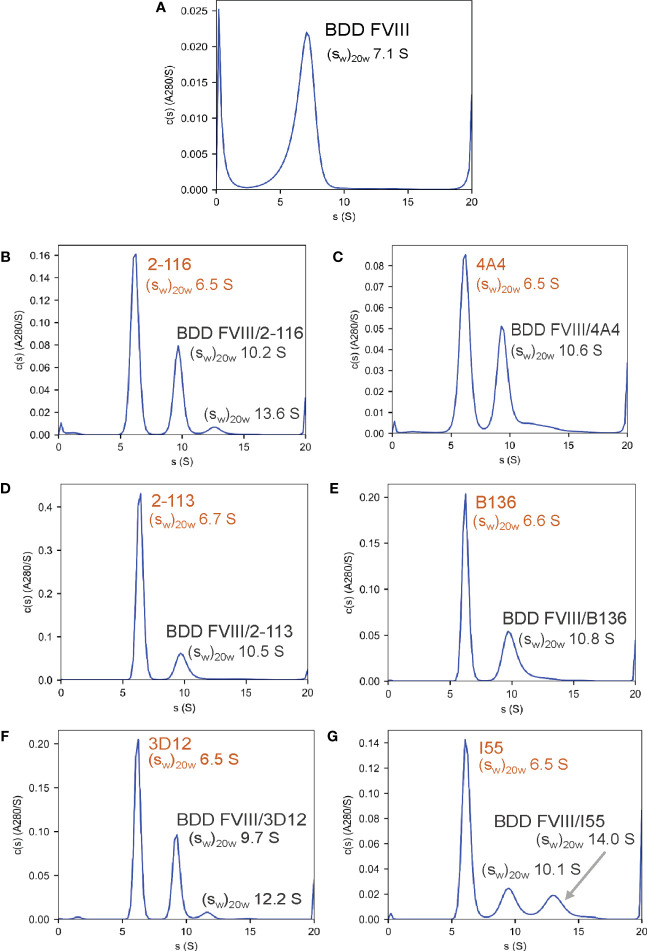
Characterization of FVIII-IC with BDD FVIII by SV AUC. Signal-average sedimentation coefficients, adjusted to the standard condition, ${\left(s_{w}\right)}_{20,w}$ , of 20°C in solvent water were estimated by integration of the continuous c(s) distributions in SEDFIT for samples containing BDD FVIII alone **(A)**, FVIII/2-116 **(B)**, FVIII/4A4 **(C)**, FVIII/2-113 **(D)**, FVIII/B136 **(E)**, FVIII/3D12 **(F)**, and FVIII/I55 **(G)** FVIII, factor VIII; FVIII-IC, FVIII-immune complexes; SV AUC, sedimentation velocity analytical centrifugation.

To confirm the formation of FVIII-IC with both FL FVIII and BDD FVIII in the setting of antibody excess, binding of a representative group of MAbs from each FVIII domain was analyzed by SV AUC. These MAbs consisted of A1 MAb 2-116, A2 MAb 4A4, A3 MAb 2-113, C1 MAb B136, and C2 MAbs 3D12 and I55. FVIII-IC samples were analyzed at 4-fold to 8-fold molar excess of MAb immediately upon thawing and sample mixing. FL FVIII and BDD FVIII in the absence of MAb produced peaks at 7.9 S and 7.1 S, respectively (**Figures 2A**, **3A**). All MAbs formed FVIII-IC with FL FVIII and BDD FVIII, inducing a shift in peaks to ~9.8–10.8 S consistent with 1:1 FVIII : MAb complex (**Figures 2B–G**, **3B–G**). FL FVIII in complex with MAbs B136, 3D12, and I55 (**Figures 2E–G**) produced broad peaks at 13–15 S suggestive of 2:1 FVIII : MAb complex. Small amounts of 2:1 FVIII : MAb complexes were also observed with BDD FVIII and MAbs 2-116, 3D12, and I55 (**Figures 3B, F, G**). Peaks at 6.4–6.7 S with FVIII-IC samples represent excess MAb (**Figures 2B–G**, **3B–G**). These results confirm the predominant formation of 1:1 FVIII : MAb complexes and small amounts of 2:1 FVIII : MAb complexes with both FL FVIII and BDD FVIII.

### Internalization of FVIII-IC is dependent on FcγRIIb and FcγRIII

**Figure 4:**
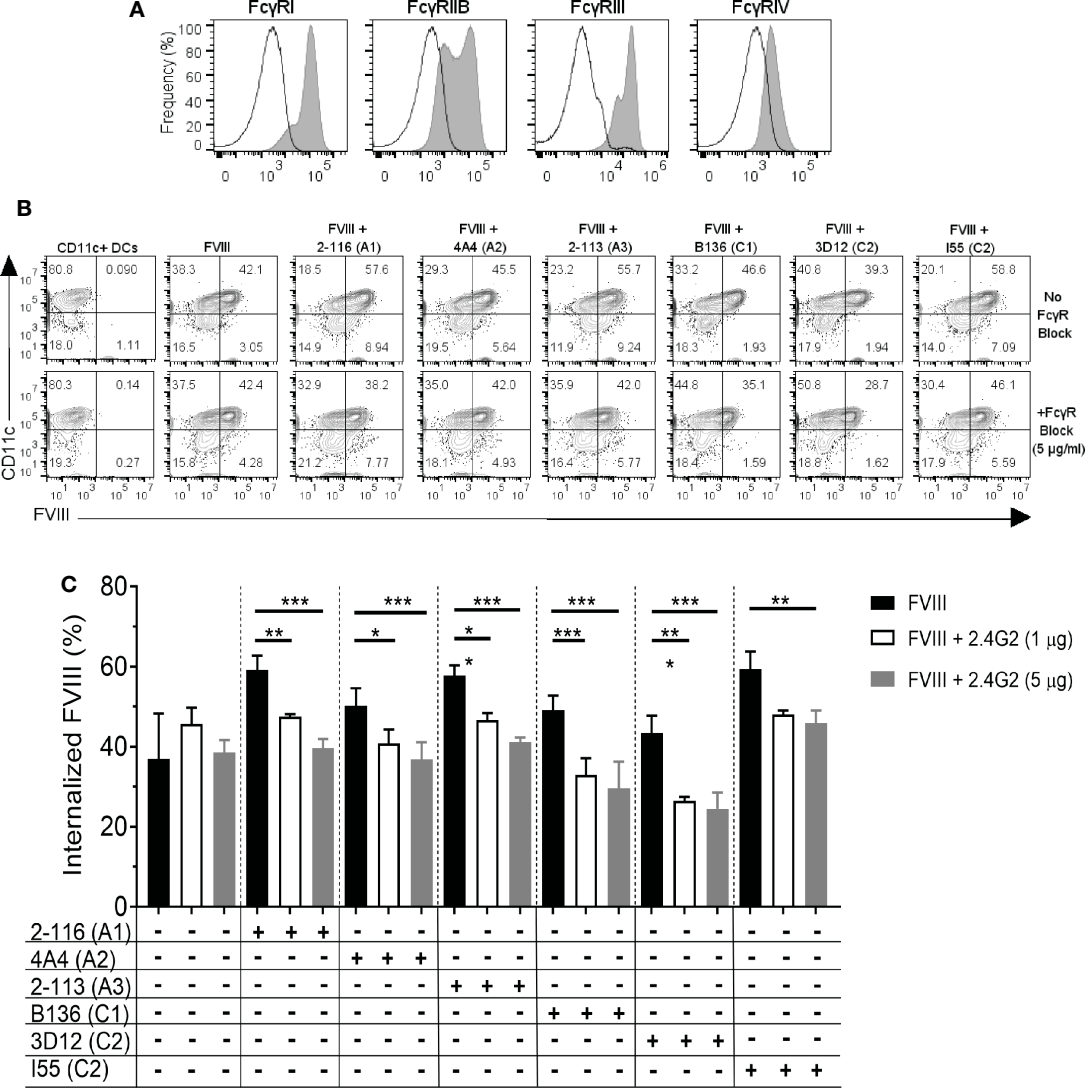
FcγRIIb and FcγRIII blockade reduces internalization of FVIII-IC by BMDC. The expression of FcγRI, IIb, III, and IV on BMDC by flow cytometry analysis is shown **(A)**. The solid black outlined histogram represents unstained and untreated BMDC, while the gray shaded histograms represent BMDC stained with the indicated FcγR. Flow contour plots of BMDC internalization of FVIII or a subset of FVIII-IC in the absence and presence of FcγRIIb/FcγRIII blockade by 5 µg/mL MAb 2.4G2 **(B)**. Summary of FVIII and FVIII-IC internalization by CD11c^+^FVIII^+^ BMDC with and without MAb 2.4G2 at 1 µg/mL and 5 µg/mL is shown **(C)**. Uptake of FVIII and each FVIII-IC group were performed in two replicate measurements in three independent experiments. Data are presented as mean ± SD. *P< 0.05, **P< 0.01, and ***P< 0.001 as determined by one-way ANOVA with Tukey’s correction for multiple comparisons. ANOVA, analysis of variance; BMDC, bone marrow-derived dendritic cells; FVIII, factor VIII; FVIII-IC, FVIII-immune complexes; MAb, monoclonal antibody.

Murine BMDC predominantly express activating FcγRI and FcγRIII in addition to inhibitory FcγRIIb (**Figure 4A**). FcγRIV is expressed on monocytes, macrophages, and neutrophils but are not highly expressed on DC (27, 28). Additionally, FcγRIIb and FcγRIII are capable of efficiently engaging and crosslinking antigen complexed with IgG1, IgG2a, and IgG2b. To evaluate the effect of FcγR blockade on FVIII-IC internalization, BMDC were incubated with a representative subset of six FVIII-IC in the presence and absence of FcγRIIb and FcγRIII blockade using MAb 2.4G2. BMDC were incubated per the manufacturer’s standard MAb concentration of 1 µg/mL and then at a 5-fold increased concentration of 5 µg/mL prior to incubation with FVIII or FVIII-IC. FVIII-IC consisting of A1 MAb 2-116, A2 MAb 4A4, A3 MAb 2-113, C1 MAb B136, C2 MAb 3D12, and C2 MAb I55 were included in these studies. Antibody-mediated blockade of FcγRIIb/FcγRIII did not affect the uptake of isolated FVIII as anticipated; however, internalization of the FVIII-IC was significantly reduced up to 35% similar to that of FVIII internalization (**Figures 4B, C**). Reduction in FVIII-IC uptake in the presence of FcγRIIb/FcγRIII blockade was not dose dependent, with similar reductions of FVIII-IC uptake observed at 1 and 5 µg/mL with the exception of FVIII/I55 (**Figure 4C**).

### FVIII^-/-^ mice immunized with FVIII-IC affect antibody responses to FVIII

To evaluate whether alterations in FVIII endocytosis by FVIII-IC observed *in vitro* would similarly affect antibody responses to FVIII *in vivo*, FVIII^-/-^ mice were immunized with FVIII or FVIII-IC. Historically, a “high-dose” FVIII regimen leads to antibody development in the majority of FVIII-immunized hemophilia A mice (42, 55). However, to be able to detect whether FVIII-IC alter the antibody response to FVIII, we initially employed a “low-dose” FVIII regimen based on previously published data demonstrating efficient immune responses with administration of lower doses of antigen when in immune complexes (14). Although determining the optimal concentration of antibody to utilize poses challenges due to multifactorial variations in antibody concentration between patients, we ultimately utilized a 1:10 FVIII-to-MAb ratio to recapitulate the degree of antibody to antigen excess in a high-titer [≥5 Bethesda units (BU)/mL] inhibitor plasma (56).

**Figure 5:**
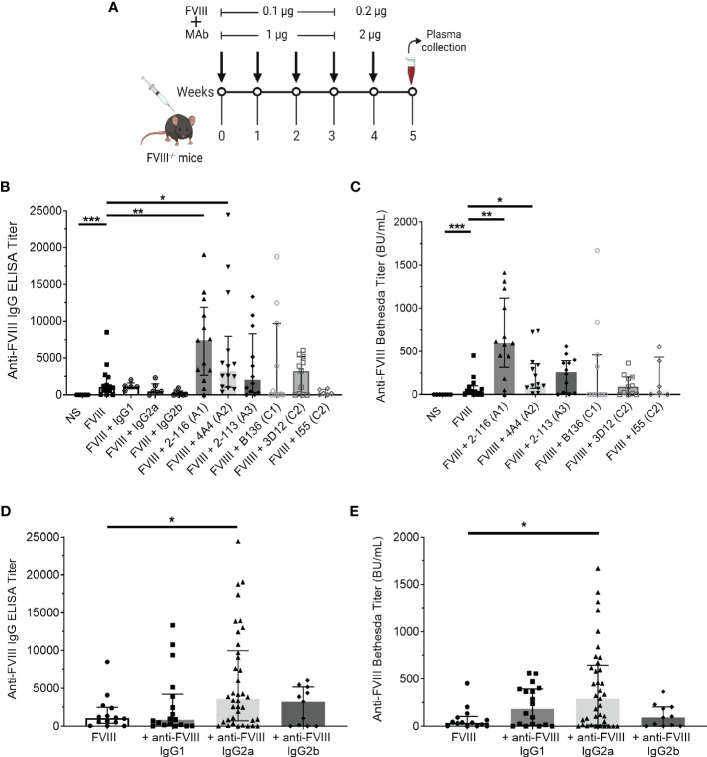
Antibody responses in FVIII^-/-^ mice immunized with FVIII or FVIII-IC using a “low-dose” FVIII regimen. Schematic of the injection regimen and plasma sampling schedule in FVIII^-/-^ mice (n = 11–14 total mice per group in three independent experiments). Injections of FVIII-IC were premixed and formed prior to injection in mice **(A)**. Median ELISA titers **(B)** and Bethesda titers **(C)** with interquartile ranges are shown. The ELISA and Bethesda titers of FVIII^-/-^ mice immunized with MAbs alone were subtracted from their respective FVIII/MAb immunization group titers to account for the potential effect of residual injected MAb in these assays. Mice injected with normal saline were negative controls. ELISA titers **(D)** and Bethesda titers **(E)** of FVIII^-/-^ mice immunized with FVIII-IC by FVIII-binding IgG isotype are shown. ELISA and Bethesda titers were compared to FVIII alone using the nonparametric Mann–Whitney U test. *P< 0.05, **P< 0.01, and ***P< 0.001. ELISA, enzyme-linked immunosorbent assay; FVIII, factor VIII; FVIII-IC, FVIII-immune complexes; MAb, monoclonal assays.

Anti-FVIII IgG ELISA titers and Bethesda titers were determined in FVIII^-/-^ mice immunized with the “low-dose” FVIII regimen consisting of 0.1 µg FVIII ± 1 µg MAb once weekly for four doses followed by a boost dose of 0.2 µg FVIII ± 2 µg MAb (**Figure 5A**). FVIII^-/-^ mice injected with FVIII/2-116 had significantly increased ELISA and Bethesda titers [median ELISA titer 7,411 BU/mL (IQR 2664, 11921) and Bethesda titer 597 BU/mL (315–1,116)] when compared to mice immunized with FVIII [ELISA and Bethesda titers: 1,063 BU/mL (402–2,476) and 37 BU/mL (2–102), respectively, P< 0.01] (**Figures 5B, C**). These results correspond to increased internalization of FVIII/2-116 by BMDC observed *in vitro* (**Figure 1B**). FVIII^-/-^ mice immunized with FVIII/4A4 had significantly increased ELISA and Bethesda titers [median ELISA and Bethesda titers 3,632 BU/mL (1,074–7,981) and 132 BU/mL (85–368), respectively] than FVIII-immunized mice. There were no significant differences in the median ELISA and Bethesda titers between FVIII^-/-^ mice injected with FVIII compared to mice injected with FVIII/2-113 [ELISA and Bethesda titers: 2,227 BU/mL (14–20) and 260 BU/mL (0–0), respectively], FVIII/B136 [ELISA and Bethesda titers: 140 BU/mL (0–9,726) and 2 BU/mL (0–459), respectively], FVIII/3D12 [ELISA and Bethesda titers: 3,249 BU/mL (20–5,184) and 92 BU/mL (0–202), respectively], or FVIII/I55 [ELISA and Bethesda titers: 1,920 BU/mL (22–760) and 37 BU/mL (0–433), respectively]. However, FVIII/B136-immunized mice trended toward reduced ELISA and Bethesda titers compared to FVIII-immunized mice. Interestingly, FVIII mice injected with FVIII/I55 had similar ELISA and Bethesda titers to FVIII-immunized mice despite increased uptake by BMDC *in vitro*, demonstrating discordance in some responses between the *in vitro* and *in vivo* assays. There were no differences in ELISA titers in mice injected with FVIII or FVIII with non-FVIII isotype controls (i.e., IgG1, IgG2a, or IgG2b), suggesting that excess IgG did not influence differences between FVIII-IC antibody titers.

Next, we analyzed median ELISA and Bethesda titers from FVIII^-/-^ mice injected with FVIII-IC by anti-FVIII MAb IgG isotype (**Figures 5D, E**). FVIII^-/-^ mice injected with FVIII-IC consisting of IgG2a had significantly higher ELISA and Bethesda titers than mice injected with FVIII alone. Compared to FVIII injections, there were no differences in ELISA and Bethesda titers between mice injected with FVIII/IgG1 or FVIII/IgG2b complexes. MAbs 2-116 and 4A4 are both IgG2a, which likely accounts for the increased antibody titers in mice immunized with IgG2a-containing FVIII-IC such as FVIII/2-116 and FVIII/4A4. However, with only one IgG2b antibody available, definitive determination of the contribution of IgG isotype on antibody responses is limited.

### Plasma antibody domain mapping of immunized mice reveals a predominance of antibodies directed against the A1 and A2 domains

**Figure 6:**
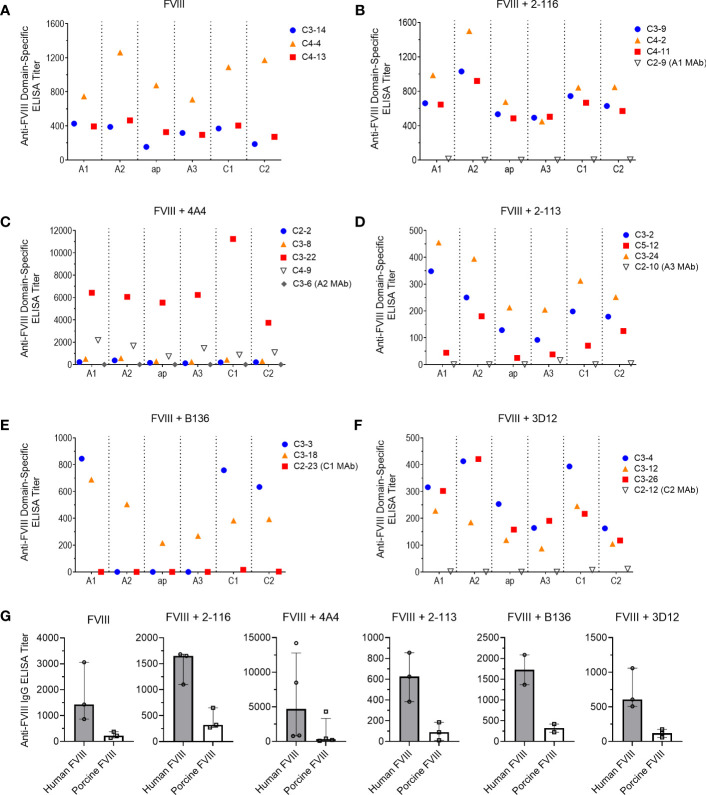
Plasma antibody domain mapping of immunized FVIII^-/-^ mice using a “low-dose” FVIII regimen. ELISA titers of *de novo* plasma antibodies against each FVIII domain, except the B domain, from FVIII^-/-^ mice (n = 3–5 mice per group) injected with FVIII **(A)**, FVIII/2-116 **(B)**, FVIII/4A4 **(C)**, FVIII/2-113 **(D)**, FVIII/B136 **(E)**, and FVIII/3D12 **(F)** are shown. Each symbol with a unique identification (e.g., C3-14, C4-13, C4-4) represents a different mouse sample. Differences in anti-FVIII IgG ELISA titers against human BDD FVIII compared to porcine BDD FVIII are shown **(G)**. BDD, B domain deleted; ELISA, enzyme-linked immunosorbent assay; FVIII, factor VIII.

To determine the effect of FVIII-IC injections over time on plasma FVIII antibody composition, we utilized a plasma domain mapping assay to evaluate domain specificity of antibodies generated by FVIII^-/-^ mice. In the six immunization groups tested, FVIII^-/-^ mice developed a polyclonal antibody response against each FVIII domain tested (**Figures 6A–F**). Mice injected with FVIII or FVIII-IC developed antibodies to human FVIII with porcine FVIII cross-reactivity (**Table 2**, **Figure 6G**). Five of the six (83%) immunization groups analyzed generated the highest titers of antibodies against the A2 domain (**Table 2**). This is consistent with prior reports describing the A2 domain as an immunodominant domain (10). Interestingly, plasma antibodies directed against the A1 domain were more frequent than antibodies against the traditionally immunodominant C2 domain in all immunization groups. The A1 domain was the second predominant domain for high ELISA titer antibody development, occurring in 67% of the FVIII-IC injection groups and in mice injected with FVIII alone. In contrast to the other FVIII-IC groups, mice injected with FVIII/2-116 had a predominance of A2 and C1 plasma antibodies, while FVIII/B136 had a predominance of A1 and C2 plasma antibodies. Mice injected with anti-FVIII MAbs without FVIII did not produce antibodies (i.e., ELISA titer ≤20) to human FVIII, porcine FVIII, or any of the isolated FVIII domains as expected (**Figures 6B–F**).

**Table 2:** Summary of plasma antibody domain mapping in immunized FVIII^-/-^ mice.

Immunization Group	Injected MAb Domain	BDD FVIII ELISA Titer*	Porcine FVIII ELISA Titer*	BDD vs. Porcine FVIII ELISA Titer P value	*De Novo* Plasma Antibodies FVIII-Binding Domain Ranking^#^
FVIII	–	1,428 [866, 3,062]	221 [136, 375]	0.10	A2 > A1 > C1 > ap> A3 > C2
FVIII + 2-116	A1	1,605 [1,102, 1,683]	323 [273, 652]	0.10	A2 > C1 > A1 > C2 > ap > A3
FVIII + 4A4	A2	4,689 [812, 12,794]	319 [128, 3,341]	0.11	A2 > A1 > C1 > C2 > ap > A3
FVIII + 2-113	A3	626 [383, 856]	89 [11, 185]	0.10	A2 > A1 > C2 > C1 > ap > A3
FVIII + B136	C1	1,727 [1,368, 2,086]	322 [225, 419]	0.33	A1 > C2 > C1> A2 = A3 = ap
FVIII + 3D12	C2	607 [508, 1,060]	120 [59, 174]	0.10	A2 > A1 > C1 > ap > A3 > C2

*Presented as median with 25th and 75th percentiles.

^#^Ranked from highest to lowest median anti-FVIII domain-specific ELISA titer.

BDD, B domain deleted; FVIII, factor VIII; MAb, monoclonal antibody.

### Antibody responses to FVIII-IC in the absence of von Willebrand factor

**Figure 7:**
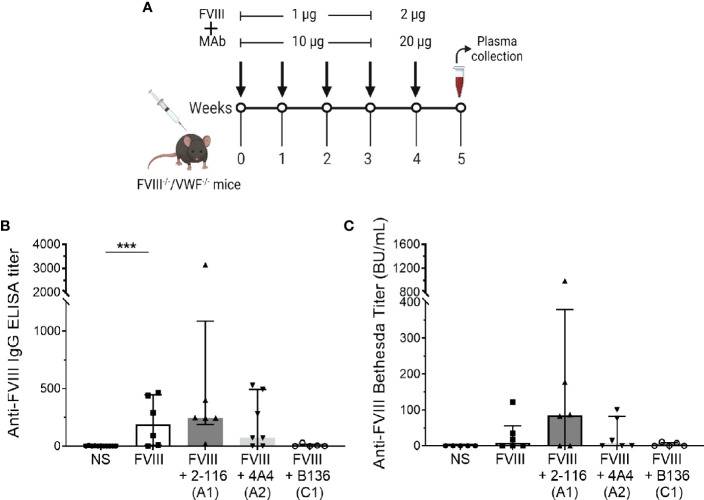
Antibody development in FVIII^-/-^/VWF^-/-^ mice injected with FVIII or FVIII-IC. Schematic of the dosing regimen in double-knockout FVIII^-/-^/VWF^-/-^ mice (n = 5–7 mice per group) injected with FVIII-IC **(A)**. ELISA titers **(B)** and Bethesda titers **(C)** of FVIII^-/-^/VWF^-/-^ mice injected with FVIII, FVIII/2-116, FVIII/4A4, and FVIII/B136 are shown. Differences in ELISA and Bethesda titers compared to FVIII were determined by the nonparametric Mann–Whitney U test and are presented as median with interquartile range. ***P< 0.001. ELISA, enzyme-linked immunosorbent assay; FVIII, factor VIII; FVIII-IC, FVIII-immune complexes; VWF, von Willebrand factor.

Von Willebrand factor (VWF) is a critical FVIII-binding ligand that reduces rapid clearance of FVIII and stabilizes FVIII in the circulation (57). To assess the role of VWF on FVIII-IC with enhanced antibody responses in FVIII^-/-^ mice (**Figure 5B**), mice deficient in both endogenous FVIII and VWF (FVIII^-/-^/VWF^-/-^ mice) were injected with FVIII, FVIII/2-116 (A1 MAb), FVIII/4A4 (A2 MAb), or FVIII/B136 (C1 MAb) using a “high-dose” FVIII regimen (**Figure 7A**). The “high-dose” regimen was employed to account for increased FVIII clearance in the absence of VWF and lower antibody titers observed in this mouse model (58). MAb B136 inhibits FVIII binding to VWF; thus, mice immunized with FVIII/B136 were included as a control. There were no differences in ELISA or Bethesda titers in FVIII^-/-^/VWF^-/-^ mice injected with FVIII, FVIII/2-116, FVIII/4A4, or FVIII/B136 (**Figures 7B, C**) despite MAbs 2-116 and 4A4 not interfering with FVIII binding to VWF (**Table 1**). These results suggest that VWF plays a role in modulating antibody responses to FVIII and FVIII-IC including MAbs that do not directly affect binding to VWF-relevant FVIII epitopes.

### In FVIII^-/-^ mice, immunization with a “high-dose” FVIII regimen suppresses differences in antibody responses of enhancing FVIII-IC regardless of antigen-to-antibody ratio

**Figure 8:**
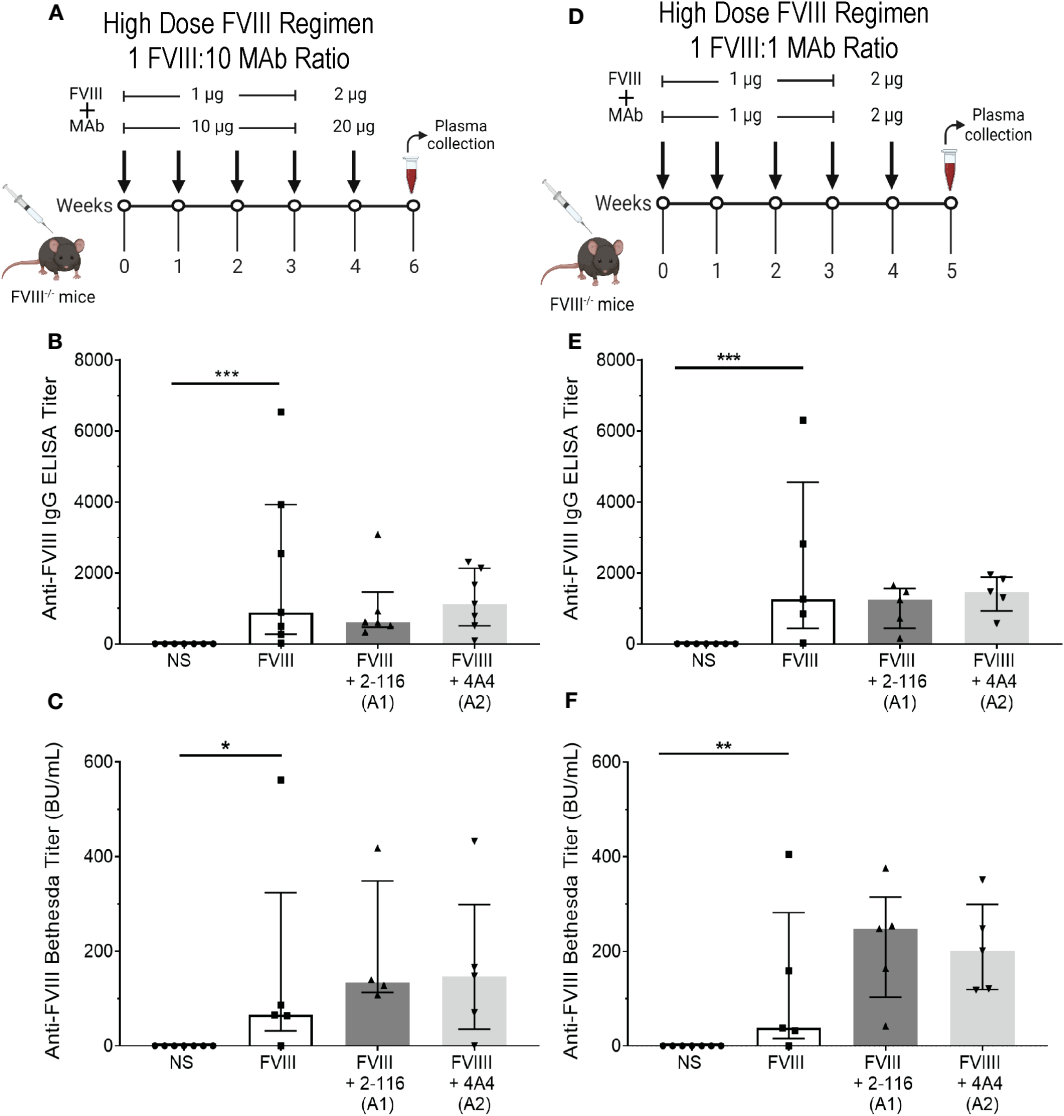
Injection of FVIII^-/-^ mice with FVIII-IC using a “high-dose” FVIII regimen masks the antibody response of enhancing FVIII/MAb complexes regardless of FVIII-to-MAb ratio. Schematic of FVIII^-/-^ mice injection regimen (n = 5–7 mice per group) and plasma sampling schedule using a high-dose FVIII regimen at a 1:10 FVIII-to-MAb ratio **(A)** or 1:1 FVIII-to-MAb ratio **(D)**. ELISA titers **(B)** and Bethesda titers **(C)** of FVIII^-/-^ mice injected with FVIII-IC at the 1:10 FVIII-to-MAb ratio dosing regimen are presented. ELISA titers **(E)** and Bethesda titers **(F)** in FVIII^-/-^ mice injected with FVIII-IC at 1:1 FVIII-to-MAb ratio dosing regimen are also shown. Differences between groups were determined by the nonparametric Mann–Whitney U test and are presented as median with interquartile range. *P< 0.05, **P< 0.01 and ***P< 0.001. FVIII, factor VIII; FVIII-IC, FVIII-immune complexes; MAb, monoclonal antibodies.

In our initial *in vivo* studies, a “low-dose” FVIII regimen consisting of 0.1 µg FVIII/1 µg MAb was utilized to account for effective immune cell activation at lower antigen doses in the context of FVIII-IC (**Figure 5A**). We hypothesized that the “low-dose” regimen would enable detection of differences in FVIII antibody responses between FVIII-IC that would have been obscured by the rapid rise in antibody titer with the use of a higher-dose FVIII regimen. To test this, FVIII^-/-^ mice were injected with a “high-dose” FVIII regimen consisting of 1 µg FVIII ± 10 µg MAb for four weekly injections followed by a boost injection of 2 µg FVIII ± 20 µg MAbs 1 week later (**Figure 8A**). There were no differences in ELISA or Bethesda titers between FVIII-, FVIII/2-116-, and FVIII/4A4-immunized mice (**Figures 8B, C**), which supports our hypothesis that a higher-dose FVIII-IC regimen masks differences in FVIII antibody responses.

Lastly, Manca et al. (59) suggested that extreme antibody excess did not alter internalization of immune complexes by APC (primarily macrophages) but could affect processing of internalized antigen and subsequent immune responses. To evaluate the role of FVIII-to-MAb ratio on antibody responses *in vivo*, we also immunized FVIII^-/-^ mice with a 1:1 ratio of FVIII to MAb consisting of 1 µg FVIII:1 µg MAb (**Figure 8D**). Similar to the “high-dose” FVIII regimen at 1:10 FVIII-to-MAb ratio (**Figures 8B, C**), there were no differences in ELISA or Bethesda titers between FVIII^-/-^ mice injected with FVIII, FVIII/2-116, or FVIII/4A4 at a 1:1 FVIII-to-MAb ratio (**Figures 8E, F**). These results suggest that the dose of FVIII-IC and not the FVIII-to-MAb ratio contributed to the antibody responses.

## Discussion

Prior studies have shown that altering or blocking C1 or C2 domain epitopes reduces FVIII endocytosis by DC (20–23). Our *in vitro* studies further demonstrate enhanced FVIII uptake by BMDC, with FVIII-IC consisting of A1 and A3 domain MAbs highlighting the contribution of these domains in FVIII immunity. We hypothesize that enhanced uptake by A1 and A3 domain MAbs may be secondary to allosteric effects upon MAb binding in which FVIII-ICs mask “protective” B-cell epitopes within the A1 and A3 domains. Alternatively, these MAbs may expose more “immunogenic” epitopes upon FVIII binding (60, 61). In these studies, epitope specificity was observed to be the primary determinant of the effect of FVIII-IC on FVIII internalization. MAb binding affinity to FVIII likely did not play a role, as the MAbs tested have nanomolar binding affinity to FVIII (23, 39–41, 44). Moreover, formation of 1:1 FVIII-IC was confirmed with both FL FVIII and BDD FVIII by SV AUC. Despite differences between FVIII-ICs on FVIII internalization by BMDC, the FVIII-IC failed to induce maturation of BMDC following a 30-min incubation. Similarly, FVIII alone did not induce BMDC activation. A danger signal or potent cosimulatory signal is necessary for T cell activation and antibody production. The absence of a danger signal or potent costimulatory signal in this study, which is typically present *in vivo*, likely accounted for the lack of DC maturation observed *in vitro* (62). However, the 30-min incubation time frame used to recapitulate the *in vitro* studies and peak FVIII-IC exposure *in vivo* may have also contributed to the lack of DC activation demonstrated.

The Fcγ receptor, specifically the inhibitory FcγRIIb (CD32), has been described as the primary mediator of endocytosis of FVIII-IC by DC and recall of FVIII-specific memory B-cell responses (19, 26, 36). In this study, FcγRIIb/FcγRIII blockade significantly reduced the uptake of FVIII-IC by BMDC derived from FVIII^-/-^ mice. In a separate study, investigators utilized a complex of FVIII and six FVIII-specific antibodies directed against the A1, A2, A3, A3-C1, and C2 domains and showed reduced internalization of the FVIII-IC by BMDC derived from wild-type C57BL/6 mice in the presence of FcγRIIb/FcγRIII blockade (19). Additional studies using various models of FcγR-deficient mice showed increased uptake of FVIII-IC by BMDC from mice lacking activating FcγRI or FcγRIII. However, increased uptake of the FVIII-IC was also observed in mice deficient in the inhibitory FcγRIIb. Vollack et al. (26) separately showed reduced formation of FVIII-specific antibody-secreting cells from immunized hemophilia A mice with FcγRIIb antibody-mediated blockade. Yet, FcγRIIb blockade did not abolish T-cell activation (63). Overall, these results suggest that internalization of FVIII-IC is dependent on the FcγR, but additional receptors or mechanisms (i.e., phagocytosis, clathrin-mediated endocytosis, or the mannose receptor) may also contribute to this process (64). Ultimately, the mechanism by which FVIII-IC alters FVIII endocytosis by APC *in vitro* and antibody responses *in vivo* warrants further exploration.

Concurrent injection of an antigen and high-affinity antiserum or antibody can induce more robust B-cell responses and T-cell proliferation against an antigen than the injection of antigen alone (14, 43, 65–67). To investigate whether changes in FVIII-IC endocytosis observed *in vitro* translated to altered antibody development *in vivo*, we similarly performed serial injections of FVIII-IC in hemophilia A mice and measured *de novo* plasma antibody titers. Notably, immunization with FVIII/A1 MAb 2-116 and FVIII/A2 MAb 4A4 enhanced antibody production in FVIII^-/-^ mice administered a “low-dose” FVIII regimen. Yet, these results were not replicated in a “high-dose” FVIII immunization regimen, suggesting that a saturation point is reached that masks differences in antibody responses in the presence of FVIII-IC. FVIII^-/-^ mice immunized with FVIII/B136 (C1 MAb) had reduced antibody titers, but this was not statistically significant. Herczenik et al. (22) separately demonstrated that pretreatment with a single dose of 1-mg human-derived C1 domain IgG1 MAb KM33 followed by 3 weeks of 1-µg FVIII injections (1,000-fold MAb-to-FVIII ratio) abolished antibody development in exon 17 knockout hemophilia A mice. However, there were no differences in antibody titers between FVIII-immunized mice pretreated with control IgG1 or KM33 after 5 weeks of injections. Our study differed from that of Herczenik et al. (22) in that we evaluated the effect of coadministration of a spectrum of FVIII-IC at a lower MAb-to-FVIII ratio on antibody responses in exon 16 knockout hemophilia A mice over time. Although there were some differences in study design, both of these studies provide evidence for epitope-dependent differences in antibody responses to FVIII-IC *in vivo*.

The concentration of antigen and antibody additionally plays a key role in inducing immune responses. One study demonstrated that OVA complexed with polyclonal anti-OVA IgG induced CD4^+^ T-cell proliferation at concentrations as low as 0.1 µg OVA (1:25 ratio of OVA to anti-IgG OVA) in contrast to 100 µg of isolated OVA necessary to induce a T-cell response *in vitro* and *in vivo* (14). Furthermore, this CD4^+^ T-cell response was dependent on DC capture of immune complexes and could not be replicated by macrophages or B cells despite FcγR expression. Manca et al. (59) reported that the degree of antibody excess encountered by APC could affect adaptive immune responses. In our study, FVIII^-/-^ mice immunized with FVIII/2-116 and FVIII/4A4 had increased antibody titers compared to FVIII alone when a lower antigen dose (0.1 µg FVIII) was utilized. This FVIII-IC enhancing effect on antibody titers was not observed when FVIII^-/-^ mice were immunized with a higher antigen dose (1 µg FVIII) regardless of FVIII-to-MAb ratio. This supports the idea that the antigen dose and not the antigen-to-antibody ratio contributed to this variability in antibody responses with FVIII/2-116 and FVIII/4A4 immunization.

Interestingly, we observed that most mice immunized with FVIII or FVIII-IC generated a predominance of antibodies that recognized the A2 domain followed by the A1 domain. FVIII/B136-immunized mice were the exception, demonstrating a predominance of A1 and C2 domain antibodies. Mice in each of these immunization groups, including mice immunized with FVIII alone, had higher ELISA titers to the A1 domain than the C2 domain. Despite a variety of domain-specific antibodies characterized in our repertoire of murine-derived anti-FVIII MAbs, the A1 domain MAb 2-116 remains the sole A1 domain MAb isolated and characterized to date (10). MAb 2-116 is a non-inhibitory IgG2a antibody that recognizes B-cell epitopes Glu11-Asp15 and Glu53-Ala78 (45). It does not affect FVIII binding to VWF, phospholipids, or thrombin activation. The predominance of A2- followed by A1-directed plasma antibodies in FVIII^-/-^ mice immunized with FVIII also differed from prior studies that identified the C2 and A2 domains as the immunodominant domains in patients and mice following FVIII exposure (8, 9). These studies primarily utilized detection antibodies against the FVIII light chain (A3-C1-C2 domains), while more recent studies have detected antibody binding to discrete domains or specific B-cell epitopes. Moreover, Healey et al. (10) demonstrated a polyclonal antibody response to FVIII inclusive of the FVIII A1 and C1 domains using the latter approach. One difference in this study that may have contributed to differences in the immunodominant domains identified was the measurement of domain-specific plasma antibody titers as opposed to B-cell hybridoma-secreted antibodies in the Healey et al. (10) study. Overall, these results support further study of the role of the A1 domain in FVIII immunity.

While the primary mechanism and receptor responsible for FVIII endocytosis by APC remain unclear, several studies have evaluated various FVIII biological properties that may influence FVIII endocytosis and ultimately FVIII adaptive immunity. Several investigators have described the protective effect of VWF on FVIII endocytosis by APC, resulting in reduced antibody production and altered memory B-cell differentiation to plasma cells (22, 68–71). Sorvillo et al. (72) showed that VWF modulates the FVIII peptides presented by MHC class II molecules and identified a repertoire of peptides that favored induction of tolerogenic CD4^+^ T-cell responses. Studies utilizing site-directed mutagenesis of C1 domain residues or epitope masking with C1 domain MAbs confirmed a role for the C1 domain in modulating FVIII uptake by DC (21–23, 73). This is further strengthened by the contribution of C1 domain residues in FVIII binding to VWF (74, 75). An international, prospective, randomized-controlled clinical trial in previously untreated patients with hemophilia A reported significant reductions in the cumulative incidence of inhibitors, including high-titer inhibitors, in children on FVIII prophylaxis with plasma-derived VWF-containing FVIII concentrates compared to recombinant FVIII products without VWF (76). Furthermore, switching to a plasma-derived FVIII/VWF concentrate is often advised in cases of failed ITI attempts with a recombinant FVIII product (77–79). In our studies, the enhanced antibody responses observed with FVIII^-/-^ mice immunized with FVIII/2-116 and FVIII/4A4 compared to FVIII alone were not observed in the absence of VWF. One would expect increased FVIII clearance in the absence of VWF to lead to decreased antigen exposure and significantly reduce immunologic responses compared to FVIII-IC. Ideally, coadministration of a FVIII/VWF product in FVIII^-/-^/VWF^-/-^ mice with FVIII-IC could provide clarity of the role of VWF in these studies. Regrettably, this approach is problematic in the hemophilia A mouse model due to the presence of human VWF in plasma-derived FVIII/VWF and recombinant VWF products, which would indelibly affect interpretation of the immune response observed due to antigenic competition (80). Although the mechanism is not yet defined, these results do suggest that VWF plays a role in antibody responses to FVIII in the absence or presence of FVIII-IC.

The standard approach to inhibitor eradication remains ITI (81). Presumably, these patients with a polyclonal mixture of antibodies form FVIII-IC with the administration of repetitive FVIII infusions during ITI. Some patients develop a robust anamnestic response upon FVIII reexposure during ITI that results in a substantial rise in the Bethesda titer (33, 77). In some cases, the Bethesda titer can peak as high as 3,000–5,000 BU/mL (76, 82, 83). These high-responding inhibitors increase the risk of severe and potentially life-threatening breakthrough bleeding symptoms and reduce the likelihood of successful ITI (34, 84). However, prior studies have shown that the antibody-binding epitope may be a stronger predictor of inhibitor pathogenicity than the Bethesda titer (58, 85). Given the lack of a murine ITI model that reflects the duration and intensity of ITI in patients, there are inherent limitations in the ability to assess the evolution of antibody development and elimination over time in murine models of hemophilia A. Moreover, this study evaluated antibody formation in naive hemophilia A mice injected with FVIII-IC in order to directly assess the impact of FVIII-IC epitope specificity. Nevertheless, these findings provide insight into a potential driver of anamnestic responses to FVIII during ITI that warrants further investigation prospectively. One group hypothesized that FVIII-IC can amplify the formation of antibody-secreting cells through the FcγRIIb and ultimately reduce ITI success (36). Fluctuations in the detection of antibodies against A2 and C2 epitopes were observed in nine patients undergoing ITI in another study, yet these antibodies persisted including in samples from individuals with inhibitor relapse (86). This provides further evidence that the effect of FVIII-IC on FVIII antibody responses is epitope-dependent and supports the development of longitudinal translational studies investigating the role of FVIII-IC on antibody responses during early FVIII exposure (<50 exposure days) as well as during and after ITI.

It is well established that patients with hemophilia A who develop inhibitors have a polyclonal response, yet the epitopes that initiate this response and the potential immune-modulatory effect of FVIII-IC have not been fully investigated. Although there are limitations in translating murine-based studies to individuals with hemophilia A and inhibitors, our study strongly suggests that FVIII-IC plays a role in the humoral response to FVIII in an epitope-dependent fashion. A better understanding of FVIII-IC in FVIII immunity could provide greater mechanistic insight into antibody development, propagation, and achievement of tolerance.

## Data availability statement

The raw data supporting the conclusions of this article will be made available by the authors without undue reservation.

## Ethics statement

The animal study was approved by Approval of animal use and study methods were granted by the Emory University Institutional Animal Care and Use Committee. The study was conducted in accordance with the local legislation and institutional requirements.

## Author contributions

GB designed and performed the experiments, analyzed the data, and wrote the article. JI, CC, EY, WB, SG, and PL conducted the experiments and acquired the data. PL and SM analyzed the data and edited the article. All authors contributed to the article and approved the submitted version.
