# Supplementary material for: Factor VIII antibody immune complexes modulate the humoral response to factor VIII in an epitope-dependent manner
Source: Front Immunol. 2023 Aug 31;14:1233356. doi: 10.3389/fimmu.2023.1233356 (PMC10501482; doi:10.3389/fimmu.2023.1233356)

**Supplemental Data**

**Factor VIII antibody immune complexes modulate the humoral response to factor VIII in an epitope-dependent manner**

Glaivy Batsuli^1,2^, Jasmine Ito^1,2^, Elizabeth S. York^1,2^, Courtney Cox^1,2^, W. Hunter Baldwin^1,2^, Surinder Gill^1,2^, Pete Lollar^1,2^, Shannon L. Meeks^1,2^*

^1^Department of Pediatrics, Emory University, Atlanta, GA, USA

^2^Aflac Cancer and Blood Disorders Center of Children’s Healthcare of Atlanta, Atlanta, Georgia, USA

***Correspondence:**

Shannon L. Meeks, M.D.

Email: smeeks@emory.edu

Supplemental content: Supplemental figure legends and figures

Supplemental figures: 2

**Supplemental Figure Legends**

**Supplemental Figure S1. MFI of FVIII uptake of a large spectrum of FVIII-IC by BMDC.** Representative flow histograms of FVIII internalization of FVIII at 37°C, FVIII at 4°C, and the 17 FVIII-IC representing each FVIII domain tested at 37°C for 30 minutes in serum-free medium are depicted. The histogram with the solid black line represents unstained BMDC as the negative control and the gray shaded histogram represents FVIII internalization by BMDC with or without MAb. The median fluorescence intensities (MFI) of FVIII at 37°C, FVIII at 4°C, and the 17 FVIII-IC are presented in each histogram.

**Supplemental Figure S2. Dendritic cell maturation does not occur with exposure to FVIII or FVIII-IC *in vitro*.** Maturation of BMDC from FVIII^-/-^ mice were analyzed using cell surface expression of CD11c, CD40, CD80/86, and MHC class II following incubation with FVIII alone or FVIII-IC at 37°C for 30 minutes in serum-free medium. Representative dot plots and histograms (bottom row) of unstained BMDC incubated with serum free medium, stained immature BMDC incubated with serum-free medium, BMDC matured with lipopolysaccharide (LPS-matured BMDC) and incubated with serum-free medium. For experimental groups, BMDC incubated with FVIII, DyLight 650-conjugated ovalbumin (OVA) as an antigen control, and two representative FVIII-IC FVIII/2-116 and FVIII/B136 are also shown. For the histograms, the solid black line represents unstained BMDC and the gray shaded histogram represents endocytosis of FVIII or OVA by BMDC.

**Supplemental Figure S1**

**
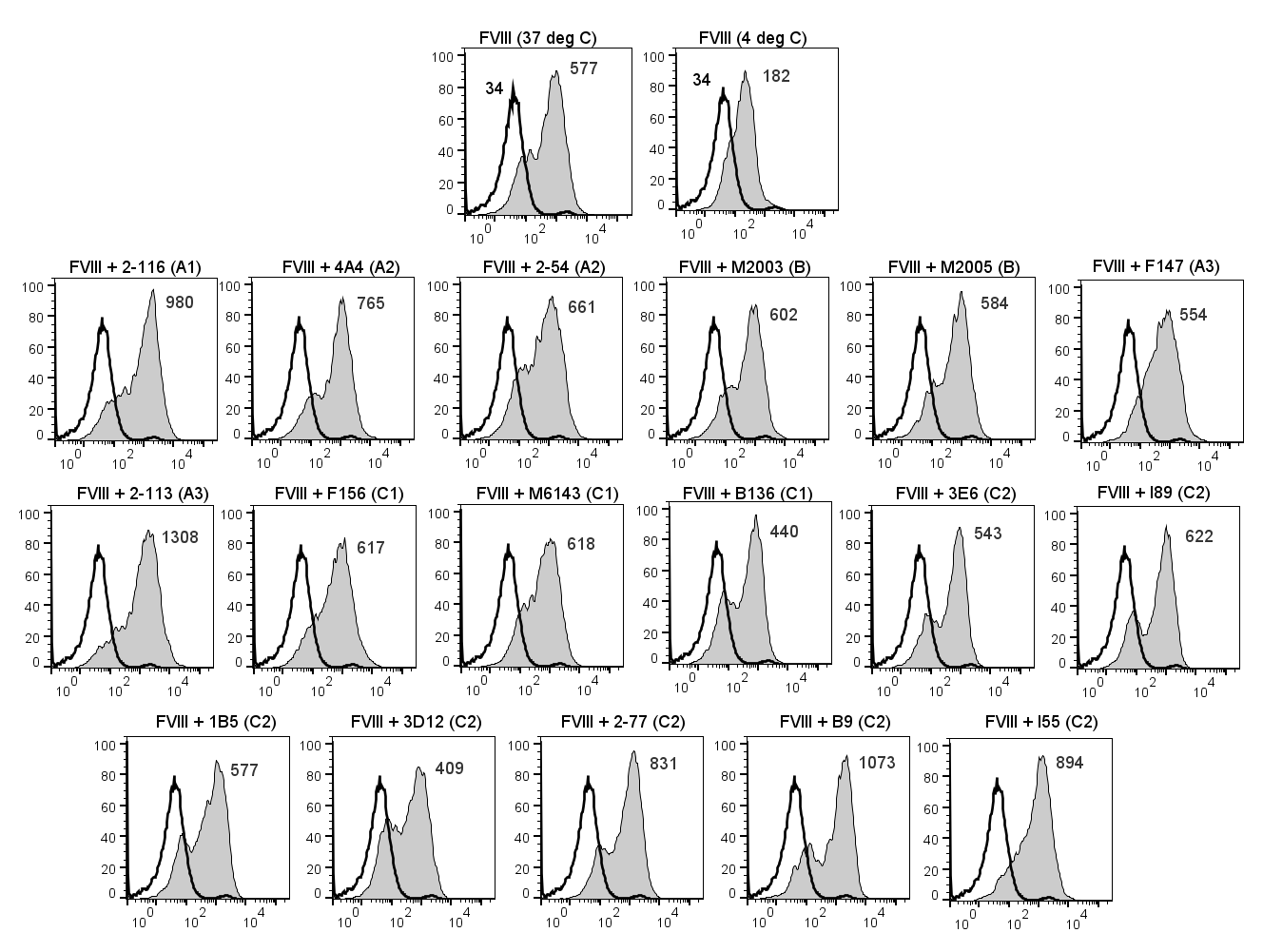
**

**Supplemental Figure S2**


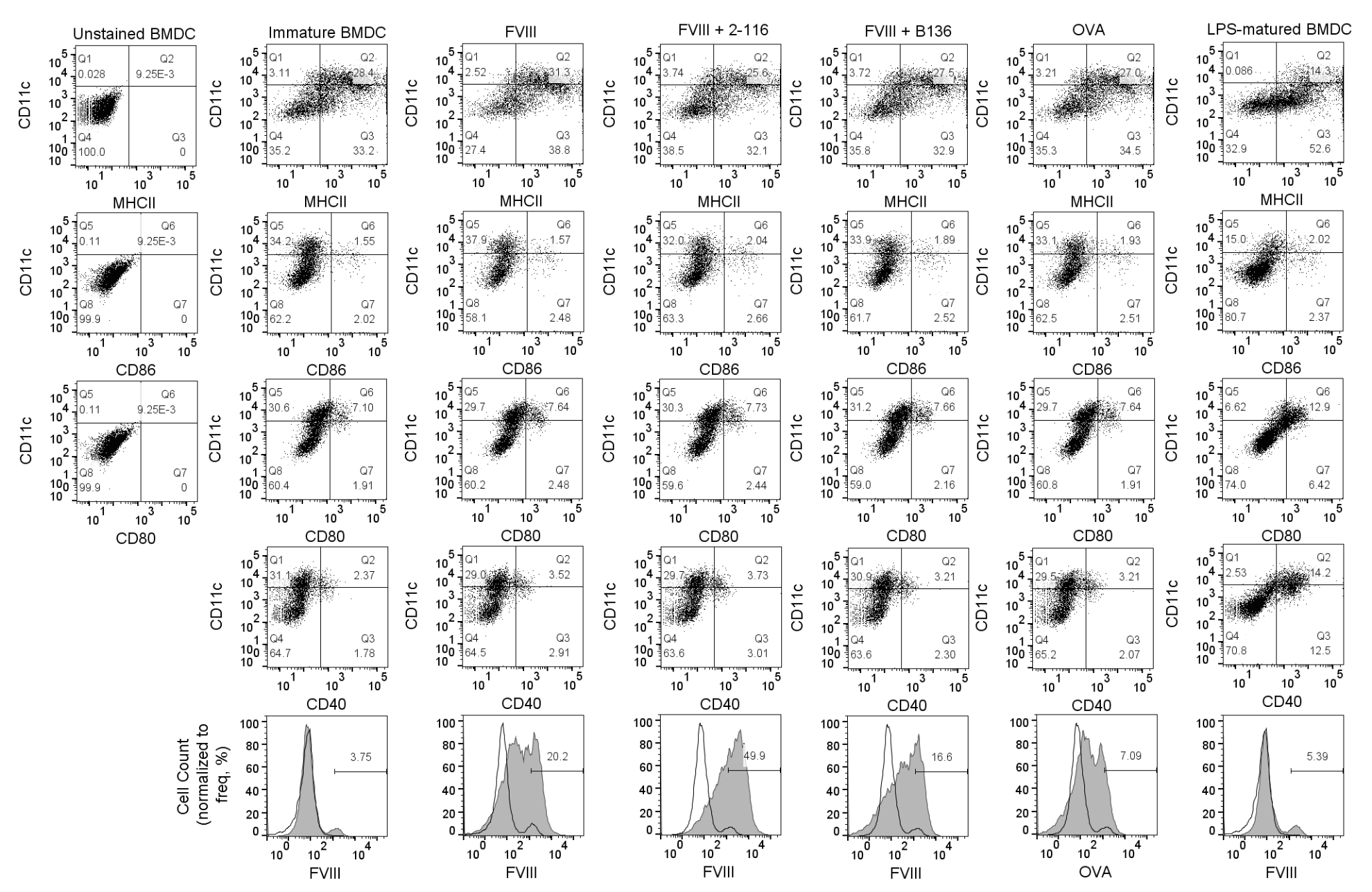

Supplement: Supplementary file 1 [file DataSheet_1.docx]
